# Study on the extraction, antioxidant and prebiotic activity of the polysaccharides from the fruits of *Phyllanthus emblica* L.

**DOI:** 10.3389/fnut.2025.1607077

**Published:** 2025-07-14

**Authors:** Haotian Huang, Xiaobo Zhou, Mengge Sun, Jiajie Chen, Tao Tan, Dongsheng Yang

**Affiliations:** ^1^School of Life Sciences, Zhuhai College of Science and Technology, Zhuhai, China; ^2^College of Life Sciences, Jilin University, Changchun, China

**Keywords:** polysaccharides, ultrasonic microwave synergistic extraction, antioxidant, probiotic, *Phyllanthus emblica* L., response surface optimization, simulated digestion

## Abstract

**Introduction:**

Polysaccharides extracted from the fruits of *Phyllanthus emblica* L. (PEP) have demonstrated various bioactivities, including antioxidant and prebiotic effects. This study aimed to optimize the extraction of PEP using ultrasonic microwave synergistic extraction (UMSE) and evaluate its bioactivities.

**Methods:**

The UMSE process was optimized using response surface methodology (RSM) to identify the most efficient extraction conditions. Antioxidant capacity was evaluated through DPPH, ABTS, and hydroxyl radical scavenging assays, alongside total reducing capacity measurements. For prebiotic activity, the ability of PEP to promote the growth of *Lactobacillus casei, Lactobacillus acidophilus*, and *Lactobacillus plantarum* was compared with that of standard prebiotics. Changes in pH and lactic acid production in the culture medium were also monitored.

**Results:**

Optimal UMSE parameters included microwave power (370 W), ultrasonic power (340 W), extraction time (25 minutes), and a solid-liquid ratio of 1:6.5 g/mL. These conditions achieved an extraction yield of 8.09%, aligning with the predicted value. The UMSE method showed higher extraction efficiency and sugar content compared to traditional water extraction, with a reduction in impurities. The extracted PEPs exhibited significant scavenging activity against DPPH, ABTS, and hydroxyl radicals, as well as robust total reducing capacity. Additionally, the PEPs demonstrated resistance to hydrolysis by artificial saliva and gastric juice, suggesting their ability to reach the gastrointestinal tract intact. In prebiotic assays, PEP (UMSE-derived, PEP-U) stimulated the proliferation of *Lactobacillus* spp. more effectively than water-extracted PEP (PEP-W), correlating with increased lactic acid production and reduced pH in the culture medium.

**Discussion:**

These results highlight the dual bioactivity of PEP as both an antioxidant and prebiotic, suggesting its potential as a functional food ingredient for promoting gut health and oxidative balance.

## 1 Introduction

*Phyllanthus emblica* L., also known as Indian gooseberry or Amla, is widely distributed across tropical and subtropical regions of Asia (1), particularly in southern Chinese provinces such as Guangdong and Yunnan, where it is extensively cultivated (2, 3). The fruit is well known not only for its nutritional richness but also for its broad-spectrum medicinal properties, including antioxidant (4, 5), anti-inflammatory (6), antimalarial (7), and antimicrobial activities (8). These health benefits are attributed to its diverse array of bioactive constituents, such as polysaccharides, flavonoids, tannins (9), and alkaloids (10).

Among these, *Phyllanthus emblica* L. polysaccharides (PEP) have attracted increasing attention due to their notable biological functions, including antioxidant (11), anti-aging, immunomodulatory (12), and anti-inflammatory effects (13–16). Recent studies also suggest their potential as prebiotic agents capable of influencing gut microbiota (17, 18). However, most existing studies focus on the pharmacological properties of PEP, with relatively limited research on the optimization of their extraction (19) and systematic evaluation of their functional activities (12), particularly their prebiotic effects.

Conventional extraction techniques—such as hot water extraction, ultrasound-assisted extraction, and enzymatic hydrolysis—are often time-consuming and inefficient (20). In contrast, ultrasonic microwave synergistic extraction (UMSE) offers a promising alternative by integrating the thermal effects of microwaves with the mechanical disruption of ultrasound (21). This method enhances extraction efficiency, shortens processing time, and reduces solvent consumption (13–16), yet its application to *Phyllanthus emblica* L. polysaccharide extraction remains underexplored.

On the other hand, maintaining gut health has become an emerging focus of functional food research (22). The gut microbiota plays a vital role in digestion, immunity, and overall health, but it is sensitive to environmental stressors, including diet and illness (23). Prebiotics—non-digestible food components that selectively stimulate beneficial gut bacteria—have been widely studied for this purpose (24). Common examples include galacto-oligosaccharides (25), inulin (26), and soybean oligosaccharides (27). However, recent literature suggests that plant-derived polysaccharides may also serve as novel prebiotics with multifunctional health benefits (28–30). For example, *Imperatae Rhizoma* polysaccharides have prebiotic properties and have the ability to promote the growth of lactic acid bacteria (31). With increasing clinical validation, polysaccharide-based prebiotics are emerging as a viable adjunctive therapy in managing chronic diseases through gut microbiota modulation (13–16, 32). Unlike traditional prebiotics, polysaccharides possess diverse structural characteristics that enable selective fermentation by distinct microbial communities, thereby offering personalized gut health benefits in clinical applications (33). Despite this, evidence regarding the prebiotic activity of *Phyllanthus emblica* L. polysaccharides remains limited and fragmented.

To address this research gap, the present study aims to: (1) optimize the extraction conditions of *Phyllanthus emblica* L. polysaccharides using UMSE, and (2) evaluate their antioxidant capacity and prebiotic potential, particularly their ability to promote the growth of *Lactobacillus* species. These findings are expected to contribute to the development of *Phyllanthus emblica*-based functional ingredients and offer new insights into the plant's gut health-promoting properties.

## 2 Materials and methods

### 2.1 Materials and reagents

Fresh *Phyllanthus emblica* L. were purchased from Guangdong, China and identified as qualified samples by Runrong Zhang. Analytically pure anhydrous ethanol, dichloromethane, n-butanol, hydrogen peroxide, and phenol were purchased from Sinopharm Chemical Reagent Co. (Beijing, China). Analytically pure glucose, glucuronic acid, and Caulmers Brilliant Blue Stain were purchased from Aladdin Biochemical Technology Co. Ltd. (Shanghai, China). Sodium tetraborate, carbazole, DPPH, salicylic acid, potassium bromide, artificial gastric fluid and artificial saliva were purchased from Shanghai Yuanye Biotechnology Co. (Shanghai, China). Concentrated sulfuric acid was purchased from Guangdong Guangzhou Chemical Reagent Factory (Guangdong, China). Ferrous sulfate, potassium ferricyanide, trichloroacetic acid, and ferric chloride were purchased from Xilong Science Co. (Guangdong, China). DNS reagent was purchased from Beijing Solarbio Technology Co. (Beijing, China).

### 2.2 Pre-processing of *Phyllanthus emblica* L.

Fresh *Phyllanthus emblica* L. fruits of uniform size and free from obvious damage and insect pests were selected and properly cleaned using ultrasonic cleaner to remove surface dust and stains. The kernels were manually removed and pulverized with a wall breaker, crushed into fine residue and placed in a freeze dryer for freeze drying process and set aside.

### 2.3 Extraction of polysaccharides

Ultrasonic microwave synergistic extraction (UMSE) (34): *Phyllanthus emblica* L. powder, pre-weighed accurately, was put into a 50 mL beaker, and dissolved in deionized water at a material-liquid ratio of 1:6.5 g/mL. The extraction process was conducted using a microwave-ultrasonic synergistic extractor for 30 min. The parameters were set as follows: microwave power of 370 W, ultrasonic power of 340 W, and temperature of 60°C .

Water extraction: Appropriate amount of *Phyllanthus emblica* L. powder was accurately weighed, added with deionized water at a material-liquid ratio of 1:6.5 g/mL, and heated in a thermostatic water bath at 60°C for 6 h.

### 2.4 Purification of polysaccharides

The extracted crude polysaccharide solution was precipitated with anhydrous ethanol in the ratio of 1:4 v/v overnight. The resulting precipitate was reconstituted with deionized water (35). Subsequently, proteins in the extract were removed using the Sevage method (chloroform: n-butanol = 4:1, v/v). This process was repeated multiple times until the proteins were completely eliminated. The extract was then dialyzed using a dialysis bag with a molecular weight cutoff of 3,500 Da for 48 h (36). Finally, the purified polysaccharide was obtained by vacuum freeze-drying. The polysaccharide derived from ultrasonic microwave synergistic extraction (UMSE) was designated as PEP-U, while the polysaccharide obtained from water extraction was referred to as PEP-W.

### 2.5 Glucose standard curve and PEP determination

The construction of the glucose standard curve was performed using the phenol-sulfuric acid method. A 0.2000 g sample of anhydrous glucose standard was accurately weighed into a beaker, dissolved, and diluted with deionized water to a final volume of 100 mL in a volumetric flask to prepare a 2 mg/mL glucose stock solution. This stock solution was sealed and stored away from light. Subsequently, 0, 0.1, 0.2, 0.4, 0.6, 0.8, 1.2, 1.6, and 2.0 mL aliquots of the 2 mg/mL glucose stock solution were taken and diluted to 10 mL with deionized water to prepare glucose sub-solutions with concentrations of 0, 0.02, 0.04, 0.08, 0.12, 0.16, 0.24, 0.32, and 0.40 mg/mL, respectively. To each sub-solution, 250 μL was taken and mixed with 125 μL of a 5% phenol solution in a fume hood. After thorough mixing, 625 μL of concentrated sulfuric acid was added. The mixtures were then heated in a water bath for 5 min, cooled to room temperature in an ice bath, and the absorbance at 490 nm was measured to plot the standard curve (37). The resulting standard curve equation was calculated as: *y* = 6.3724*x* + 0.0016, *R*^2^ = 0.9997.

Following the completion of the reaction, the PEP solution was diluted 20-fold. A 250 μL aliquot of the diluted polysaccharide solution was then mixed with 125 μL of phenol solution and 625 μL of concentrated sulfuric acid solution. This mixture was heated in a water bath for 5 min and then cooled to room temperature in an ice bath. The reaction solution of polysaccharide was spectrally scanned within the wavelength range of 400–1,000 nm using a UV spectrophotometer. It was observed that a pronounced absorption peak appeared at 490 nm, indicating that this wavelength represents the maximum absorption for the quantification of PEP content. The absorbance at 490 nm was utilized to calculate the polysaccharide content using the following formula:


(1) $$\text{Polysaccharide}\;\text{content}\;(\text{\%})\text{=}\frac{\text{C}\times \text{V}\times \text{D}\times 10^{-3}}{\text{M}}\times 100$$

where *C* is the concentration of the polysaccharide solution (mg/mL); *V* is the total volume of the solution (mL); *D* is the number of dilutions and *M* is the mass of the sample (g).

The formula for the polysaccharide extraction rate is:


(2) $$\begin{array}{l} \text{Polysaccharide}\;\text{extraction}\;\text{rate}\;(\text{\%})=\frac{{\text{M}}_{\text{PEP}}}{{\text{M}}_{\text{Phyllanthus emblica L}.}} \end{array}$$

where *M*_*PEP*_ is the weight of polysaccharide weighed after extraction (g); *M*_*Phyllanthus emblica L*._ is the weight of sample added by extraction (g).

### 2.6 Protein content determination

Determination of protein content is essential for elucidating the purity of polysaccharides (38). The protein content was determined using the Coomassie brilliant blue assay. Aliquots of 0, 0.01, 0.02, 0.04, 0.06, 0.08, and 0.1 mL of a standard protein solution were prepared, each brought to a final volume of 0.1 mL with water, followed by the addition of 6 mL of Coomassie brilliant blue G-250 solution. The mixtures were thoroughly mixed, allowed to stand for 10 min, cooled to room temperature and then the absorbance was at 595 nm. These measurements were used to plot the standard curve, which was found to have the equation: *y* = 0.0924*x* + 0.1707, *R*^2^ = 0.9988.

### 2.7 Determination of alduronic acid content

The content of alduronic acid was determined by carbazole-sulfuric acid method (39). A series of seven standard solutions with glucuronic acid concentrations of 0%, 10%, 20%, 30%, 40%, 50%, and 100% were prepared. After cooling in an ice water bath, 1 mL of sodium tetraborate-sulfuric acid solution was added to each standard solution. The solutions were then heated in a boiling water bath for 5 min, followed by cooling in an ice water bath at room temperature. Subsequently, 0.04 mL of carbazole-ethanol solution was added to each solution, mixed uniformly, and then its absorbance value was measured at the wavelength 523 nm, with distilled water was used as the blank control. The standard curve of glucuronic acid was plotted with glucuronic acid content as the horizontal coordinate (*x*) and absorbance value as the vertical coordinate (*y*). The standard curve equation for glucuronic acid content was calculated as: *y* = 0.002*x* + 0.103, *R*^2^ = 0.9995.

### 2.8 Single-factor experimental design

Single-factor experiments offer foundational insights into the individual effects of specific variables and facilitate a more profound understanding of their interactions and impacts (35). The extraction of polysaccharide using UMSE is influenced by several factors, including microwave power, ultrasonic power, extraction time, and material-liquid ratio. A single-factor experimental design was utilized to elucidate the influence of these experimental factors on polysaccharide yield, it the identification of the most influential factor guiding subsequent RSM investigations. Deionized water was chosen as the solvent in these experiments, which assessed the impact of varying the microwave power (100, 200, 300, 400, 500 W), ultrasonic power (100, 200, 300, 400, 500 W), extraction times (10, 15, 20, 25, 30 min), and feed-to-liquid ratios (1:2, 1:4, 1:6, 1:8, 1:10 mL/g).

### 2.9 Response surface methodology design

Response surface methodology (RSM) is a statistical technique employed to ascertain the optimal experimental conditions that yield desired response variable values (40). By constructing a response surface model and using it for optimization, the most favorable combination of factor levels can be de-identified, thereby enhancing the extraction process parameters in a manner that is both environmentally sustainable and economically efficient. Following the analysis of aforementioned single-factor experiments, the factors of microwave power (*A*), ultrasonic power (*B*), extraction time (*C*), and material-liquid ratio (*D*) were selected as the independent variables for RSM. The extraction rate of PEP (*Y*) was designated as the dependent variable. A four-factor, three-level experimental model was established using Box-Behnken central composite design. The optimal PEP extraction process was determined by fitting the quadratic equation that models the relationship between each factor and the response variable. This approach allows for the identification of the interaction effects and the determination of the optimal conditions for maximizing PEP extraction efficiency.

### 2.10 Monosaccharide composition

Referenced methods for determining monosaccharide composition and modified them (13–16). Specifically, a measured quantity of polysaccharide sample was introduced into an ampoule and solubilized in 1 mL of distilled water. Subsequently, 1 mL of 4 M trifluoroacetic acid (TFA) was added. The ampoule was sealed and subjected to hydrolysis at 110°C for 4 h in an oven. After hydrolysis, the solution was neutralized with NaOH. A 200 μL aliquot of the neutralized solution was mixed with 200 μL of an internal standard. Subsequently, 100 μL of the mixture was combined with 100 μL of 0.3 M NaOH and 0.5 M PMP, and incubated in a water bath at 70°C under light protection for 1 h. Upon cooling to ambient temperature, 100 μL of 0.3 M HCl was added to neutralize the solution. The resulting mixture underwent multiple chloroform extractions prior to HPLC analysis. The HPLC analysis was conducted under the following conditions: an Agilent Eclipse XDB-C18 column (5 μm, 4.6 × 250 mm) was utilized; the flow rate was 1 mL/min; the column temperature was maintained at 30°C; and detection was performed at a wavelength of 254 nm. The mobile phase consisted of solution A (0.4% TEA in 18% acetonitrile) and solution B (0.4% TEA in 60% acetonitrile), employing a gradient elution technique.

### 2.11 Infrared analysis

A 400 mg sample of KBr was weighed and baked in oven until moisture was removed. From this dried batch, 200 mg of KBR was taken and ground into a fine, white powder. This powder was then pressed and placed in IR detector for background processing (13–16). Subsequently, 200 mg of the dried KBr was combined with 2 mg of polysaccharide powder. The mixture was thoroughly ground to achieve a homogeneous fine powder. This powder was pressed into a tablet, which was then scanned using an IR spectrometer over the wavenumber range of 4,000–400 cm^−1^ (41).

### 2.12 Scanning electron microscopy analysis

The sample was secured onto a specimen mount using conductive adhesive and subsequently coated with a layer of gold via sputtering. Subsequent examination was performed using scanning electron microscopy (SEM) at magnifications of 1,000× and 10,000× (13–16).

### 2.13 Antioxidant activity analysis

#### 2.13.1 Determination of DPPH radical scavenging ability

DPPH free radical scavenging activity experiments were performed using a slightly modified version of previously reported protocols (42). A 2 mL of DPPH-ethanol solution (0.1 nmol/mL) was combined with 2 mL of polysaccharide solution at concentrations of 0.2, 0.4, 0.6, 0.8, 1.0 mg/mL. The mixtures were allowed to react for 30 min in a dark environment, after which the absorbances were measured at 517 nm. Vitamin C served as a positive control in this experiment. The DPPH clearance rate was calculated using the following formula:


(3) $$\begin{array}{l} \text{DPPH}\;\text{radical}\;\text{scavenging}\;\text{activity}\;(\text{\%})=\left(1-\frac{{\text{A}}_{\text{sample}}-{\text{A}}_{\text{sblank}}}{{\text{A}}_{\text{control}}-{\text{A}}_{\text{cblank}}}\right)\\ \;\times 100\text{\%} \end{array}$$

where *A*_*sample*_ is the absorbance of a mixture of DPPH and sample, *A*_*sblank*_ is the absorbance of a mixture of sample and ethanol, *A*_*control*_ is the absorbance of a mixture of solvent and DPPH without sample, and *A*_*cblank*_ is the absorbance of a mixture of solvent and ethanol without sample.

#### 2.13.2 Determination of ABTS radical scavenging ability

ABTS free radical scavenging activity experiments were performed using a slightly modified version of previously reported protocols (43). A solution of 2.45 mmol/L potassium persulfate was blended with ABTS working solution (7 mmol/L) in the dark at room temperature and then stored overnight. The ABTS radical solution was then diluted with sodium phosphate buffer (pH 7.4) to attain an absorbance of 0.70 ± 0.02 at 734 nm. Subsequently, 1.5 mL of this working solution was combined with 0.5 mL of the test sample. The absorbance at 734 nm was recorded after a 6-min reaction period. Vitamin C was employed as a positive control in this assay. ABTS clearance rate was calculated as the following formula:


(4) $$\begin{array}{l} \text{Radical}\;\text{scavenging}\;\text{activity}\;(\text{\%})=\left(1-\frac{{\text{A}}_{1}-{\text{A}}_{2}}{\text{A}}\right)\times 100\text{\%} \end{array}$$

Where *A* is the negative control, *A*_1_ is the absorbances of the reaction solution and *A*_2_ is the blank control.

#### 2.13.3 Determination of hydroxyl radical scavenging ability

The method for determining hydroxyl radical scavenging activity was modified slightly from previous protocols (44). To 150 μL of polysaccharide solutions with concentrations of 0.2, 0.4, 0.6, 0.8, and 1.0 mg/mL, 25 μL of FeSO_4_ (1.5 mM), 10 μL of a salicylic acid-ethanol solution (20 nM), and 15 μL of hydrogen peroxide were added. The mixtures were then thoroughly mixed and incubated in a water bath at 37°C for 60 min. Using distilled water as blank control and Vitamin C as positive control, the hydroxyl radical scavenging activity was assessed by measuring the absorbance at 510 nm and was calculated using formula (3).

#### 2.13.4 Reducing power assay

A volume of 100 μL of samples with varying concentrations was combined with 200 μL of a 1% potassium ferricyanide solution and 200 μL of phosphate buffer solution (pH 6.6). The mixture was thoroughly agitated and incubated at 50°C for 30 min. After cooling, 200 μL of 10% trichloroacetic acid solution was introduced, and the mixture was centrifuged at 10,000 rpm for 5 min. The supernatant (400 μL) was then combined with 80 μL of 0.1% FeCl_3_ solution and 400 μL of deionized water, and the resulting mixture was allowed to react for 10 min. The absorbance of the solution was subsequently measured at 700 nm. Ascorbic acid was used as a positive control in this assay. Reducing power was calculated as follows:


(5) $$\begin{array}{l} \text{Reducing}\;\text{power}={\text{A}}_{\text{i}}-{\text{A}}_{\text{j}} \end{array}$$

Among them, *A*_*i*_ is the absorbance of the sample mixed with FeCl_3_ and *A*_*j*_ is the absorbance of the blank control without FeCl_3_.

### 2.14 Simulated digestion of polysaccharides *in vitro*

#### 2.14.1 Effect of artificial saliva on polysaccharide hydrolysis

The hydrolysis behavior of PEP-U and PEP-W in artificial saliva was evaluated (45). Fructooligosaccharides (FOS) was used as a positive control. The pH of the artificial saliva was adjusted to 5, 6, 7, and 8 using 1 M HCl and sodium phosphate, respectively. Subsequently, PEP-U, PEP-W, and FOS, each at a concentration of 100 mg, were dissolved in artificial saliva at the respective pH levels to achieve a volumetric concentration of 1.0% (w/v), and the solutions incubated at 37°C for 6 h. Samples were taken at intervals of 0, 1, 2, 4, and 6 h to assess the release of reducing sugars. The samples were further incubated for additional 5 h at 37°C . The content of reducing sugar was quantified using the DNS method (46), while total sugar was determined with the phenol-sulfuric acid method mentioned. The hydrolysis degrees of samples were calculated according to the formula as follows:


(6) $$\begin{array}{l} \text{Hydrolysis}\;\text{degree}\;(\text{\%})\text{ = }\frac{\text{reducing}\;\text{sugar}\;\text{released}}{\text{total}\;\text{sugar}-\text{initial}\;\text{reducing}\;\text{sugar}}\times 100\text{\%} \end{array}$$

Where reducing sugar released was the difference between the reducing sugar content at specified time and the initial reducing sugar content.

#### 2.14.2 Effect of artificial gastric juice on polysaccharide hydrolysis

Evaluate the hydrolysis sensitivity of PEP-U and PEP-W in artificial gastric juice using existing research methods (47). FOS was used as a positive control. The pH of the artificial gastric fluid was adjusted to 1, 2, 3, 4, and 5, respectively, using 1 M HCl. For each pH level, an equivalent volume of buffer was added to the sample solution, and the reaction mixtures were incubated at 37°C for 6 h. Aliquots were collected at 0, 1, 2, 4, and 6 h to monitor the hydrolysis progress. The reaction mixture was then incubated for 6 h. The reaction mixture was then incubated for 6 h at 37°C. The reducing sugar content was quantified using the DNS method (46), while the total sugar content was determined with the phenol-sulfuric acid method mentioned. The hydrolysis degrees of samples were calculated according to the formula (6).

### 2.15 Effect of polysaccharides on the growth of probiotics

PEP-U, PEP-W and FOS were added to the MRS sugar-free medium at mass concentrations of 0, 0.5, 1, 1.5, 2, and 3% (w/v). Subsequently, three strains of probiotic bacteria, *Lactobacillus casei, Lactobacillus acidophilus* and *Lactobacillus plantarum*, were individually inoculated into the medium and incubated with shaking at 37°C for 36 h. The OD_600nm_ of the cultures was measured across the varying polysaccharide concentrations to ascertain the optimal polysaccharide supplementation level (48).

FOS was used as a positive control in the experiment. Optimal concentrations of polysaccharide, PEP-U, PEP-W and FOS, were added to the MRS sugar-free medium. Subsequently, three strains of probiotics (*Lactobacillus casei, Lactobacillus acidophilus* and *Lactobacillus plantarum*) were introduced and incubated for 36h. The OD_600nm_ of the culture solution was measured at 0, 2, 4, 8, 12, 18, 24, 30, and 36 h. Additionally, the lactic acid content and pH variations were assessed at regular intervals. Lactate content was quantified using a colorimetric assay involving NAD^+^ as the hydrogen receptor. Lactate dehydrogenase (LDH) catalyzed the conversion of lactate to produce pyruvate, concomitantly reducing NAD^+^ to NADH. Phenazine methosulfate (PMS) mediated the transfer of hydrogen to nitro blue tetrazolium (NBT), treading to the formation of a purple-colored complex, which was then quantified by measuring the absorbance at 530 nm (49). The dynamic change of pH was measured using a pH meter.

### 2.16 Statistical analysis

In this study, each experiment was repeated three times and results are reported as mean ± standard deviation. Response surface experiments were designed using Design-Expert 13 software. Plots were completed using Origin 2021. Statistical analysis was performed using one-way analysis of variance (ANOVA). *p* < 0.05 was used to define statistical significance.

## 3 Results and discussion

### 3.1 Analysis of one-way experiment

#### 3.1.1 Effect of microwave power on the extraction rate of polysaccharides

**Figure 1:**
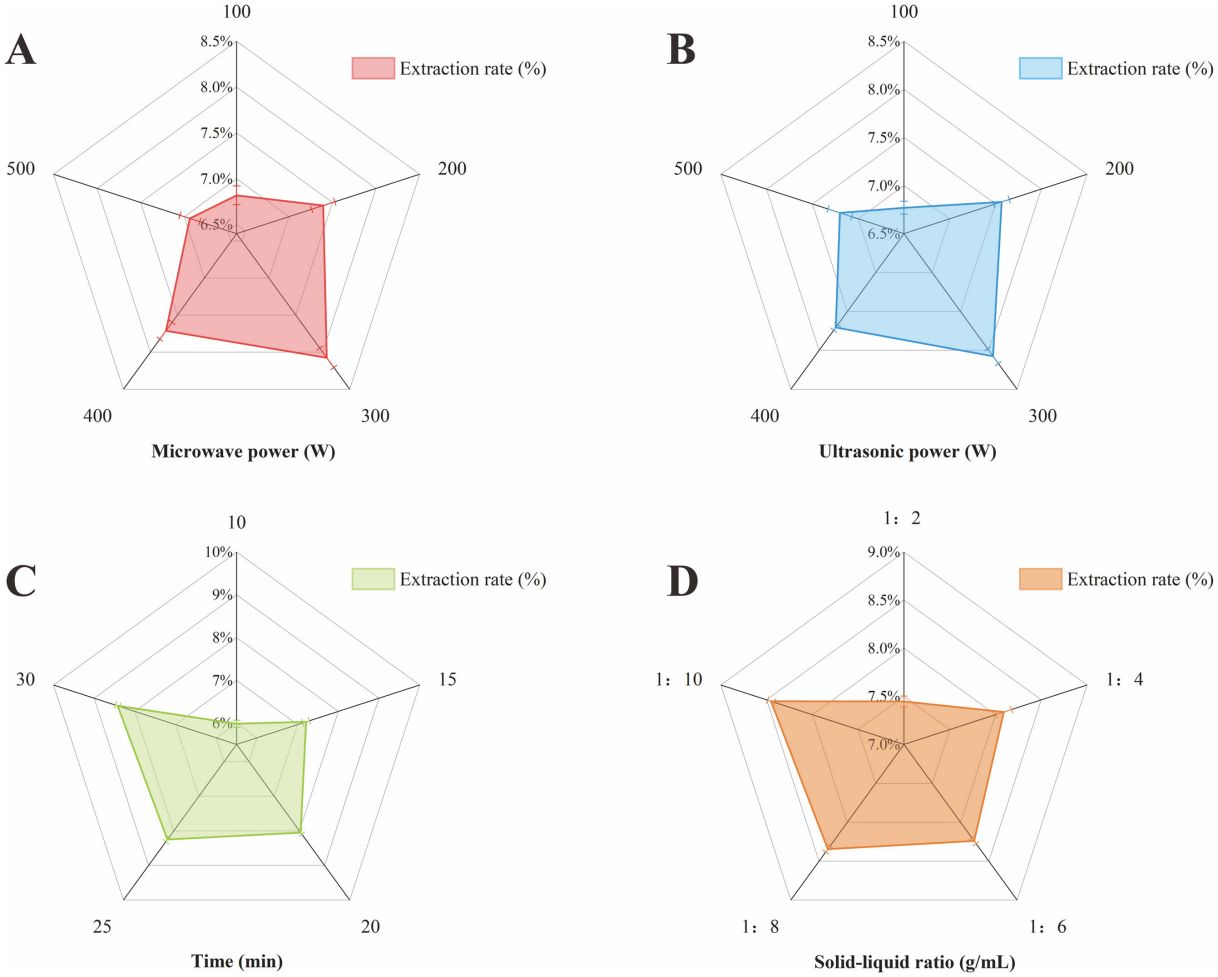
Effect of each single factor on polysaccharide extraction rate. **(A)** Microwave power. **(B)** Ultrasonic power. **(C)** Time. **(D)** Material-liquid ratio.

Microwave energy expedites the dissolution and diffusion processes of polysaccharides and can disrupt cell wall structure, thereby facilitating the release of intracellular polysaccharides and enhancing extraction efficiency (50). Figure 1A shows the effect of microwave power on polysaccharide extraction rate. Within the range of 100–300 W, the polysaccharide extraction rate escalated with increasing microwave power, achieving a peak at 300 W. Beyond this threshold, the extraction rate declined, likely due to excessive microwave power causing structural damage or degradation of the polysaccharides. Hence, a microwave power of 300 W is deemed the optimal process parameter for polysaccharide extraction, as it maximizes extraction efficiency without compromising the integrity of the polysaccharide structure.

#### 3.1.2 Effect of ultrasonic power on polysaccharide extraction rate

Ultrasonic energy, through its vibrational effects, facilitates the rapid penetration of solvent into the herbal material, thereby enhancing dissolution of the active constituents (51). Figure 1B illustrates the impact of ultrasonic power on the rate of polysaccharide extraction. As the ultrasonic power increases, the polysaccharide extraction rate also incrementally rises. However, at power levels below 300 W, the vortex and bubble motion within the medium are insufficiently vigorous, leading to a diminished ultrasonic effect. Conversely, when the ultrasonic power exceeds 300 W, the high-density vortices and potent bubble activity can alter the polysaccharide structure, potentially resulting in the loss of active ingredients (52). Given these observations, an ultrasonic power of 300 W is identified as the optimal parameter for polysaccharide extraction. This level balances the enhancement of extraction efficiency with the preservation of polysaccharide integrity, avoiding the degradation of active components that could occur at higher power settings.

#### 3.1.3 Effect of extraction time on polysaccharide extraction rate

Generally, an extended extraction time correlates with enhanced polysaccharide extraction efficiency, as it allows for more thorough contact between the solvent and the sample, facilitating the dissolution and diffusion of polysaccharide into the solvent. The extraction rate typically increases rapidly at the outset but may decelerate as time progresses. Overly prolonged extraction times can lead to cross-linking or aggregation of polysaccharide molecules, which can impede the extraction rate (53). Figure 1C delineates the effect of extraction time on PEP extraction rate. The data indicate a rapid increase in the polysaccharide extraction rate when the extraction time was between 10 and 20 min, suggesting that shorter extraction time are insufficient for optimal polysaccharide extraction. Beyond 20 min, the rate of increase in polysaccharide extraction rate becomes marginal, indicating that the majority of polysaccharides have been extracted at this point. Excessive extraction times can significantly diminish economic viability and are thus not conducive to industrial large-scale extraction (54). Consequently, an extraction time of 25 min is determined to be the optimal parameter for polysaccharide extraction, balancing extraction efficiency with economic considerations.

#### 3.1.4 Effect of feed-liquid ratio on polysaccharide extraction rate

Figure 1D demonstrates the impact of the material-liquid ratio on the extraction rate of polysaccharide. The extraction rate of polysaccharide peaked at a material-liquid ratio of 1:8 g/mL. Below this ratio, specifically at <1:6 g/mL, the polysaccharide extraction rate increased sharply with the increments in the material-liquid ratio. Conversely, beyond a 1:6 g/mL, the rate of increase in polysaccharide extraction rate slowed down, and further increases in the material-liquid ratio led to heightened solvent usage and associated costs. Therefore, the material-liquid ratio of 1:6 g/mL is identified as the optimal parameter for polysaccharide extraction, balancing extraction efficiency with solvent economy.

### 3.2 Response surface experimental design analysis

To determine the optimal parameters for the four variables under investigation, a response surface design was implemented using Design-Expert 13, following the outcomes of a preliminary one-way experiment. The specific parameters of response surface design are shown in Table 1. The resulting mathematical regression model, which predicts the polysaccharide extraction rate as a function of microwave power (*A*), ultrasonic power (*B*), extraction time (*C*), and material-liquid ratio (*D*), is formulated as follows:


$$\begin{array}{ll} Y=8.31\;+\;0.0948A+0.4851B+0.2623C+0.1334D\\ - & 0.3205AB-0.4291AC+0.022AD+0.29BC+0.533BD\\ - & 0.136CD-0.8537A^{2}-0.5562B^{2}-0.5946C^{2}-0.62D^{2}\; \end{array}$$

**Table 1:** Response surface experiment factor level table.

**Level**	**Microwave power (W) A**	**Ultrasonic power (W) B**	**Time (min) C**	**Material- liquid ratio (mL/g) D**
−1	200	200	20	1:50
0	300	300	25	1:65
1	400	400	30	1:80

In the context of RSM, the *F*-value serves as a metric for evaluating the goodness of fit of the response surface model. A substantial *F*-value suggests that the factors and interaction terms within the model effectively account for the variation the response variable (29, 30). This indicates a closer alignment between the response surface model and actual data, thereby offering a more precise depiction of the system's behavior. The *p*-value associated with the lack-of-fit term is employed to assess the model's adequacy in representing the actual data. A low *p*-value (typically <0.05) signifies a good fit, indicating that the model closely mirrors the empirical data. On the other hand, a high *p*-value suggests a notable discrepancy between the model's predictions and the actual data, implying that the model may not be adequately capturing the nuances of the real-world scenario and thus may not be an appropriate descriptor of the system under study.

As presented in Tables 2–4, the *F*-value for the model was 68.17 (*p* < 0.01) and the *p*-value of the misfit term was 0.7342 (*p* > 0.05) indicating that the mathematical regression model of the extraction rate of polysaccharides was highly significant, with low error, less influenced by unknown factors, and the model was valid and of high research significance. The *R*^2^ was 0.9855, the Adjusted *R*^2^ was 0.9711, both values were close to 1, with a difference of 0.0144. The coefficient of variation (C.V.) was 1.69%, further suggesting that the model accounts for the majority of the total variance, demonstrating the model's good reliability and high precision. Therefore, this mathematical regression model can be selected as a reference for studying polysaccharide extraction rate, optimizing process parameters, and predicting extraction rate.

**Table 2:** Response surface methodology (RSM) design options and results (*n* = 29).

**Number**	**A**	**B**	**C**	**D**	**Extraction rate (%)**
1	200	200	25	6	6.00240
2	400	200	25	6	6.79514
3	200	400	25	6	7.56564
4	400	400	25	6	7.07652
5	300	300	20	4	6.55827
6	300	300	30	4	7.47707
7	300	300	20	8	6.90511
8	300	300	30	8	7.28006
9	200	300	25	4	6.58276
10	400	300	25	4	6.69211
11	200	300	25	8	6.93357
12	400	300	25	8	7.13109
13	300	200	20	6	6.72842
14	300	400	20	6	7.18124
15	300	200	30	6	6.55427
16	300	400	30	6	8.16726
17	200	300	20	6	6.09185
18	400	300	20	6	7.21388
19	200	300	30	6	7.47093
20	400	300	30	6	6.87659
21	300	200	25	4	7.07551
22	300	400	25	4	6.96480
23	300	200	25	8	6.34020
24	300	400	25	8	8.36139
25	300	300	25	6	8.47871
26	300	300	25	6	8.25975
27	300	300	25	6	8.40391
28	300	300	25	6	8.12646
29	300	300	25	6	8.29783

**Table 3:** ANOVA table for regression model of PEP extraction rate.

**Source**	**Sum of squares**	**d*f***	**Mean square**	***F*- value**	***p*- value**	**Significance**
**Model**	14.22	14	1.02	68.17	<0.0001	^**^
A	0.1080	1	0.1080	7.24	0.0176	^*^
B	2.82	1	2.82	189.44	<0.0001	^**^
C	0.8255	1	0.8255	55.39	<0.0001	^**^
D	0.2136	1	0.2136	14.33	0.0020	^**^
AB	0.4108	1	0.4108	27.56	0.0001	^**^
AC	0.7365	1	0.7365	49.41	<0.0001	^**^
AD	0.0019	1	0.0019	0.1304	0.7234	–
BC	0.3365	1	0.3365	22.58	0.0003	^**^
BD	1.14	1	1.14	76.24	<0.0001	^**^
CD	0.0739	1	0.0739	4.96	0.0428	^*^
*A* ^2^	4.73	1	4.73	317.17	<0.0001	^**^
*B* ^2^	2.01	1	2.01	134.61	<0.0001	^**^
*C* ^2^	2.29	1	2.29	153.88	<0.0001	^**^
*D* ^2^	2.49	1	2.49	167.30	<0.0001	^**^
**Residual**	0.2087	14	0.0149			
Lack of fit	0.1351	10	0.0135	0.7342	0.6859	–
Pure Error	0.0736	4	0.0184			
**Cor Total**	14.43	28				

“^**^” is highly significant (p < 0.01); “^*^” is significant (p < 0.05); “–” is not significant.

**Table 4:** Reliability analysis of PEP extraction rate regression model.

**Project**	**Numerical value**	**Project**	**Numerical value**
Std. Dev.	0.1221	*R* ^2^	0.9855
Mean	7.23	Adjusted *R*^2^	0.9711
C.V. %	1.69	Predicted *R*^2^	0.9381
		Adeq Precision	26.3118

In the primary terms, factor *B, C*, and *D* exhibit high significance, while factor A demonstrates significant. Regarding the interaction terms, AB, AC, BC and BD are highly significant, CD is significant, and AD is not significant. In the secondary terms, A^2^, B^2^, C^2^ and D^2^ are all highly significant. Based on the magnitude of the *F*-value associated with the primary terms, the influence of those factors on the polysaccharide extraction rate can be ranked in descending order: Ultrasonic power (*B*) > Extraction Time (*C*) > Material-liquid ratio (*D*) > Microwave power (*A*). This ranking suggests that ultrasonic power has the most pronounced effect on the extraction rate, followed by extraction time, material-liquid ratio, and microwave power, respectively.

**Figure 2:**
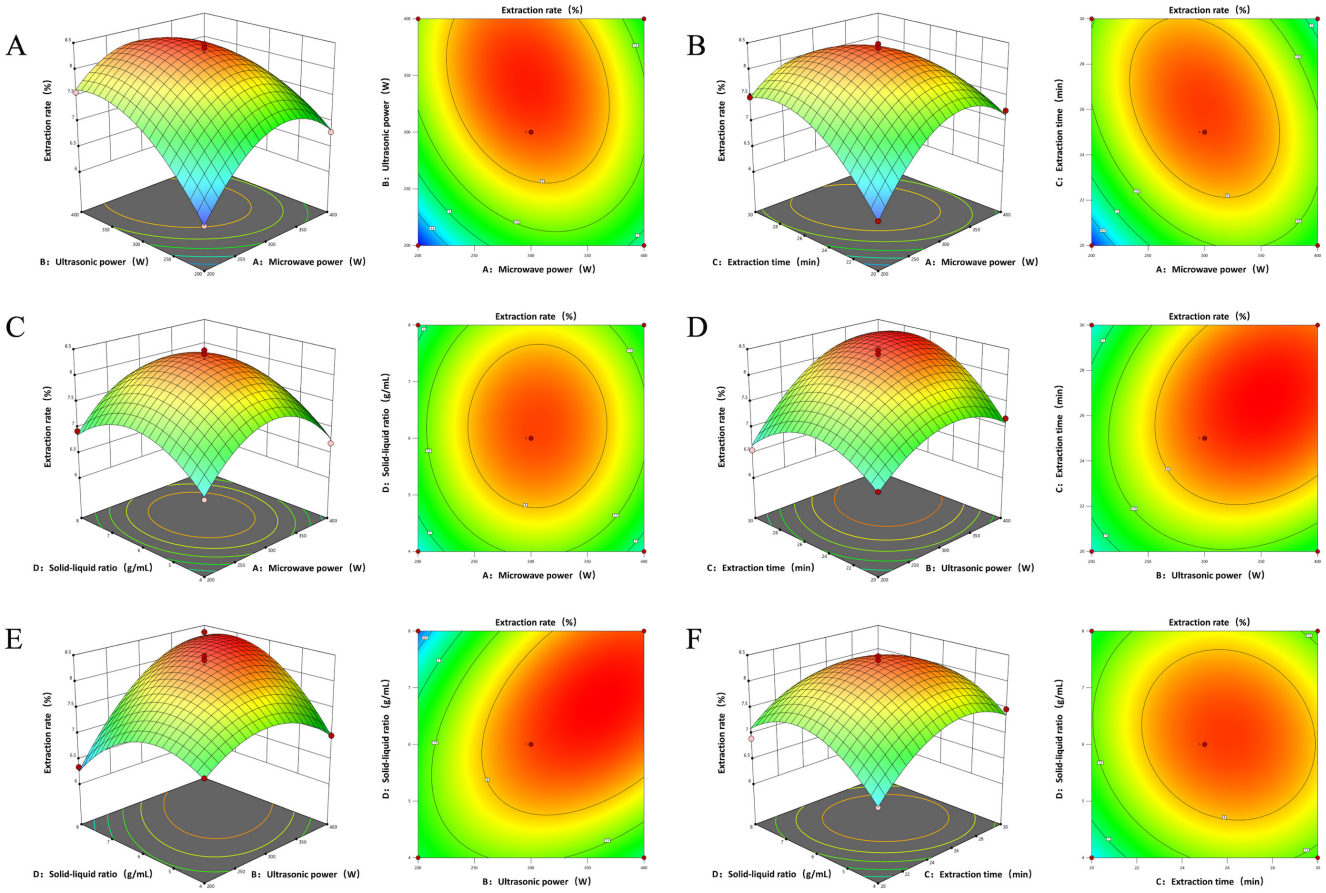
3D interaction and contour plots of the effect of interaction between two factors on polysaccharide extraction rate **(A)** Microwave power and ultrasound power. **(B)** Microwave power and time. **(C)** Microwave power and material-liquid ratio. **(D)** Ultrasonic power and time. **(E)** Ultrasonic power and material-liquid ratio. **(F)** Time and material-liquid ratio.

Within the response surface methodology (RSM) framework, three-dimensional (3D) factor interaction plots are crucial for predicting the relationship between factors and extraction rates, as shown in Figure 2. The contour lines within these plots serve as indicators of the interaction strength between factors (55). Specifically, an elliptical shape of the contour lines suggests a significant interaction between the two factors, whereas a circular shape indicates a lack of significant interaction. The gradient of the response surface's factor interaction 3D plot is indicative of the influence each factor exerts on the polyelectrolyte (PEP) extraction rate; a steeper slope corresponds to a more pronounced effect of the factor on the extraction rate, and conversely, a gentler slope suggests a less significant impact. Additionally, the rate of color change within the contours directly mirrors the importance of each factor on the response value, providing a visual assessment of their relative significance in the extraction process (56).

The interaction between microwave power (*A*) and ultrasonic power (*B*) was found to be highly significant, as evidenced by the clear elliptical contour lines, indicating a pronounced synergistic effect between these two variables. The extraction rate increased sharply with escalating microwave and ultrasonic powers, followed by a gradual decline after peaking, suggesting a broad optimal range for these parameters. This is consistent with the analysis of variance (ANOVA) results of the established mathematical regression model. The synergistic action of ultrasound's mechanical effects and microwave's thermal effects is conducive to the more efficient disruption of plant cell walls, thereby facilitating the release of polysaccharides. Additionally, the rapid internal heating by microwaves augments the temperature gradient across the cell membrane, which in turn enhances the cavitation effects of ultrasonic waves, leading to improved extraction efficiency. This indicates that the integrated application of microwave and ultrasonic energy in polysaccharide extraction can significantly shorten the extraction times and enhance productivity (2).

The interaction between microwave power (*A*) and extraction time (*C*) was found to be significant, with a contour plot exhibiting a distinct elliptical shape. The extraction rate of PEP demonstrated an initial increase followed by a decrease as both microwave power and extraction time increased. Notably, the slope and range of the extraction rate in relation to extraction time were more pronounced than those associated with microwave power, suggesting that extraction time exerts a more substantial influence on the extraction rate of PEP than the microwave power, suggesting that extraction time exerts a more substantial influence on the extraction rate of polysaccharides than microwave power. This finding aligns with the results obtained from the ANOVA of the mathematical regression model. During the extraction process, both the extraction time and microwave power affect the transfer rate of active substances between the solvent and the sample. Within an optimal range, these two factors synergistically enhance the extraction efficiency. As extraction time and microwave power increase, additional microwave energy is absorbed by the sample, which accelerates the internal temperature rise and the diffusion of bioactive substances, consequently leading to an increase in the PEP extraction rate (57).

The interaction between the ultrasonic power (*B*) and extraction time (*C*), notably significant, as evidenced by the 3D response surface plots, which exhibited a pronounced upward-convex shape, and the contour lines, which revealed an elliptical pattern (58). Within an optimal range of ultrasonic power, prolonging the extraction time can increase the yield of PEP. However, beyond a certain threshold, the rate of increase in extraction may diminish. Utilizing ultra-high power or excessively long extraction times could potentially degrade the polysaccharides, thereby reducing the extraction efficiency. Consequently, it is imperative to select an appropriate combination of extraction time and ultrasonic power to maximize the extraction yield of PEP without compromising their integrity.

The contour plots depicting the interaction effects between ultrasonic power (*B*) and material-liquid ratio (*D*) showed an ellipse shape, while the response surface exhibited a convex shape, suggesting a significant interaction with a discernible maximum value. The extraction of polysaccharides initially increased with the augmentation of both ultrasonic power and material-liquid ratio, followed by a gradual decline. The impact of ultrasonic power on polysaccharide extraction rate was markedly more pronounced than that of material-liquid ratio, a finding consistent with the ANOVA from the mathematical regression model. The augmentation of ultrasonic power induces ultrasonic oscillation within the material-liquid, facilitating the rupture of cell walls. Concurrently, the intensity of acoustic pressure waves within the solvent increases, leading to the rupture of bubbles in the solvent and the generation of violent liquid convection. These phenomena enhance the motion at the material-liquid interface and the diffusion rate of the biologically active substances within the liquid phase, thereby augmenting the polysaccharide extraction rate.

The contour plots between microwave power (*A*) and feed to liquid ratio (*D*), extraction time (*C*) and feed to liquid ratio (*D*) were approximately circular with high *P*-values, comparing with lower correlations between the other groups.

The optimal parameters for the extraction of PEP were determined using the mathematical regression model derived from Design-Expert 13, which yielded the following: microwave power of 368.844 W, ultrasonic power of 338.052 W, extraction time of 25.163 min, material-liquid ratio of 1:6.558 g/mL, and a predicted polysaccharide extraction rate of 8.045%. However, considering instrumental limitations and practical constraints, the parameters were adjusted to microwave power of 370 W, ultrasonic power of 340 W, extraction time of 25 min, and material-liquid ratio of 1:6.5 g/mL for practical application. To validate these optimized conditions, five replication experiments were carried out. The results, shown in Table 5, demonstrate that the polysaccharide extraction rates from these validation experiments closely matched the predicted values, with an RSD = 1.8672% < 2.00%. This low RSD indicates that the response surface optimization method is highly reproducible and reliable, and thus, the optimized parameters are suitable for the PEP extraction process.

**Table 5:** Results of PEP extraction rate validation experiments (*n* = 5).

**Number**	**Extraction rate (%)**	**RSD (%)**
1	8.1455	1.8672
2	7.9061	
3	8.1789	
4	7.9659	
5	8.2666	

### 3.3 Effect of the two extraction methods on polysaccharide

To assess the differences in the composition of the extracts obtained by the two extraction methods, we quantified the sugar, protein and glucuronic acid contents of the polysaccharides obtained by ultrasonic microwave synergistic extraction (UMSE) and conventional water extraction. As shown in Table 6, there were significant differences in the composition of the products obtained by the different extraction methods. Compared to water extraction, the UMSE method achieves a higher polysaccharide extraction rate, sugar content (reaching 78.21%), and glucuronic acid content, while simultaneously resulting in a lower protein content. The UMSE method utilizes the synergistic effects of mechanical and thermal actions to enhance extraction efficiency (59). Ultrasound disrupts plant cell walls to promote the heating of intracellular substances by microwaves, thereby increasing polysaccharide yields (60). Water extraction is less efficient in comparison, as it relies solely on heat to disrupt the structure of the cell.

**Table 6:** Composition of polysaccharides obtained by different extraction methods.

**Polysaccharide species**	**Yield (%)**	**Sugar content (%)**	**Protein content (%)**	**Glucuronic acid content (%)**
PEP-U	8.0926	78.21	12.22	12.08
PEP-W	7.6433	59.34	20.34	10.74

Ultrasound degrades non-carbohydrate components such as proteins and other impurities (61), while the UMSE method selectively extracts carbohydrate-rich components (62), thereby increasing the sugar content of the final extract. UMSE methods may result in degradation or denaturation of certain proteins due to strong physical and thermal effects (4, 5). The decrease in protein content in UMSE also shows that certain proteins are still captured or broken down into smaller fragments. In contrast, water extraction co-extracts a wider range of non-sugar substances such as proteins or secondary metabolites leading to a decrease in sugar content.

In terms of energy efficiency and scalability, the combination of ultrasound and microwave energy in the UMSE method is more effective in disrupting the plant matrix, which in turn promotes the release of acidic polysaccharide constituents (63). This synergy may make UMSE more scalable for industrial applications. Water extraction, on the other hand, is a relatively mild treatment, so it may face efficiency bottlenecks in large-scale production. Therefore, the UMSE method is more efficient in releasing glucuronic acid, which is a key component in polysaccharide studies.

### 3.4 Monosaccharide composition

The monosaccharide compositions of PEP-W and PEP-U are detailed in Table 7. For PEP-W, the composition was as follows: Galacturonic acid (GalA) 28.05%, Glucose (Glc) 25.97%, Galactose (Gal) 31.69%, and Xylose (Xyl) 14.29%. In the case of PEP-U, the composition comprised Mannose (Man) 6.22%, Rhamnose (Rha) 5.45%, GalA 12.43%, Glc 44.45%, Gal 11.39%, and Xyl 20.07%. Notably, Gal was the predominant monosaccharide in PEP-W, while Glc was the most abundant in PEP-U. Galactose was predominant in PEP-W, but the percentage of Glucose was higher in PEP-U. Such differences may be closely related to the different extraction methods and the exerted physical effects. These treatments may have caused different monosaccharides to have different release effects during the disruption of cell wall structure, ultimately resulting in different proportions of them in the final extract.

**Table 7:** Analysis of the monosaccharide composition of PEP-U and PEP-W [Mass percent (%)].

**Polysaccharide species**	**Man**	**GlcN**	**Rha**	**GlcNAc**	**GlcA**	**GalA**	**Glc**	**Gal**	**Xyl**	**Fuc**
PEP-U	0	0	0	0	0	28.05	25.97	31.69	14.29	0
PEP-W	6.22	0	5.45	0	0	12.43	44.45	11.39	20.07	0

### 3.5 FT-IR analysis

**Figure 3:**
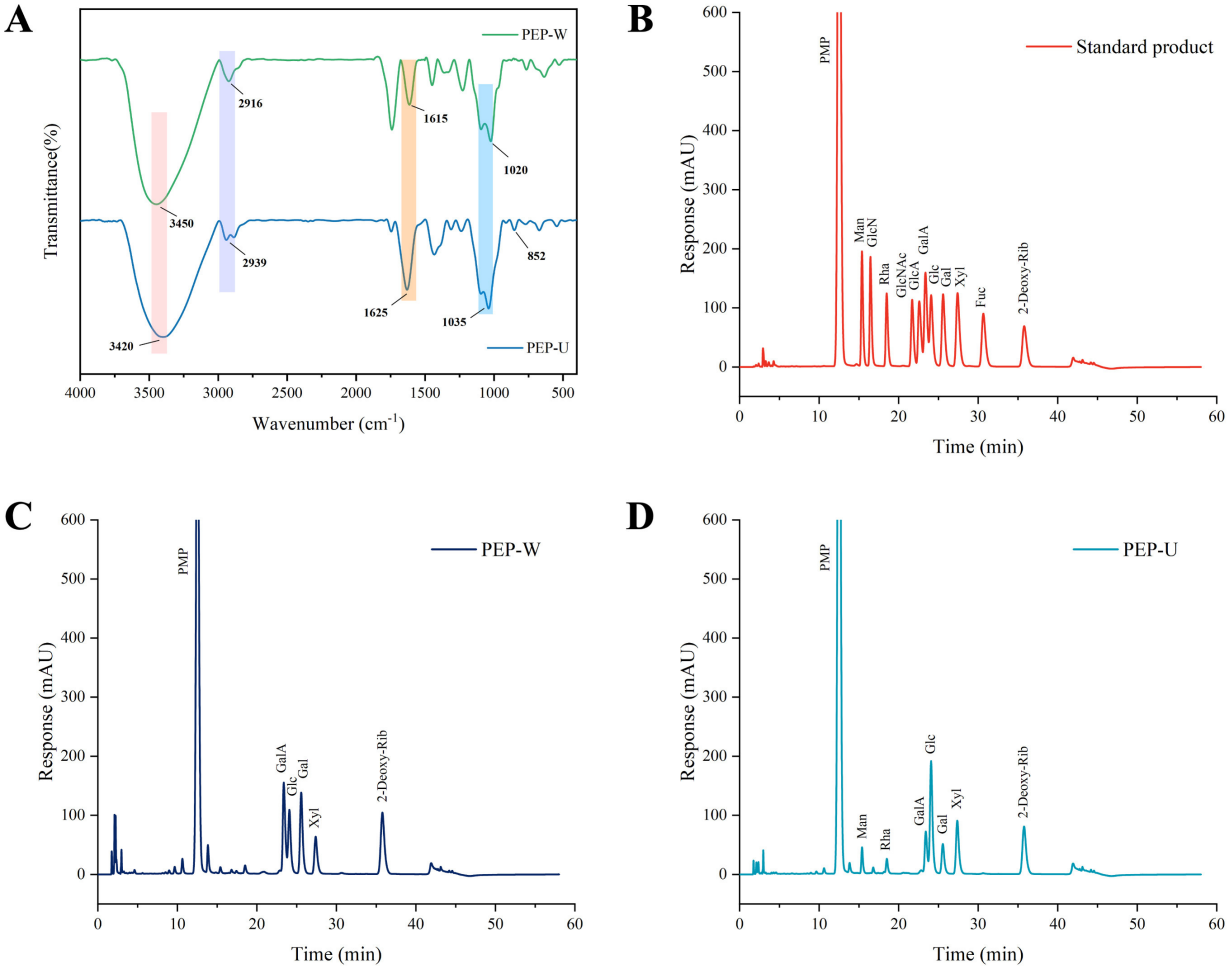
Structural characterization of PEP-W and PEP-U. **(A)** Infrared spectra of PEP-W and PEP-U. **(B)** Standard for monosaccharide composition. **(C)** Monosaccharide composition of PEP-W. **(D)** Monosaccharide composition of PEP-U.

Fourier transform infrared spectroscopy was used to observe the functional groups of PEP-U and PEP-W within the wavenumber the range of 4,000–400 cm^−1^. As shown in Figure 3, prominent absorption peaks at 3,450 and 3,420 cm^−1^ could be assigned to hydroxyl O-H bond stretching vibrations (4, 5). The weak peaks observed near 2,916 and 2,939 cm^−1^ correspond to the stretching vibration of the C-H bond. The absorption peaks at 1,615 and 1,625 cm^−1^ indicated the presence of glycosuronic acid. Peaks at 1,020 and 1,035 cm^−1^ are attributed to the asymmetric stretching vibration of the C-O-C bond, indicative of the pyranose ring structure in both polysaccharides. Furthermore, the characteristic absorption peak appearing at 852 cm^−1^ is indicative of the α-glycosidic bond in PEP-U (64). These characteristic absorption peaks confirm the presence of typical polysaccharide functional groups in both PEP-U and PEP-W. However, PEP-U differed from PEP-W in structure especially in the absorption peak of α-glycosidic bond (852 cm^−1^). This difference may be closely related to the disruptive effect of ultrasound and microwave treatments on the cell wall, which further supports the higher porosity and fragmented structure of PEP-U observed in the SEM results. All of the above demonstrates that the different extraction methods, in addition to differing in the composition of the obtained monosaccharides, also have a significant effect on the functional groups of the molecular structure.

### 3.6 SEM results analysis

**Figure 4:**
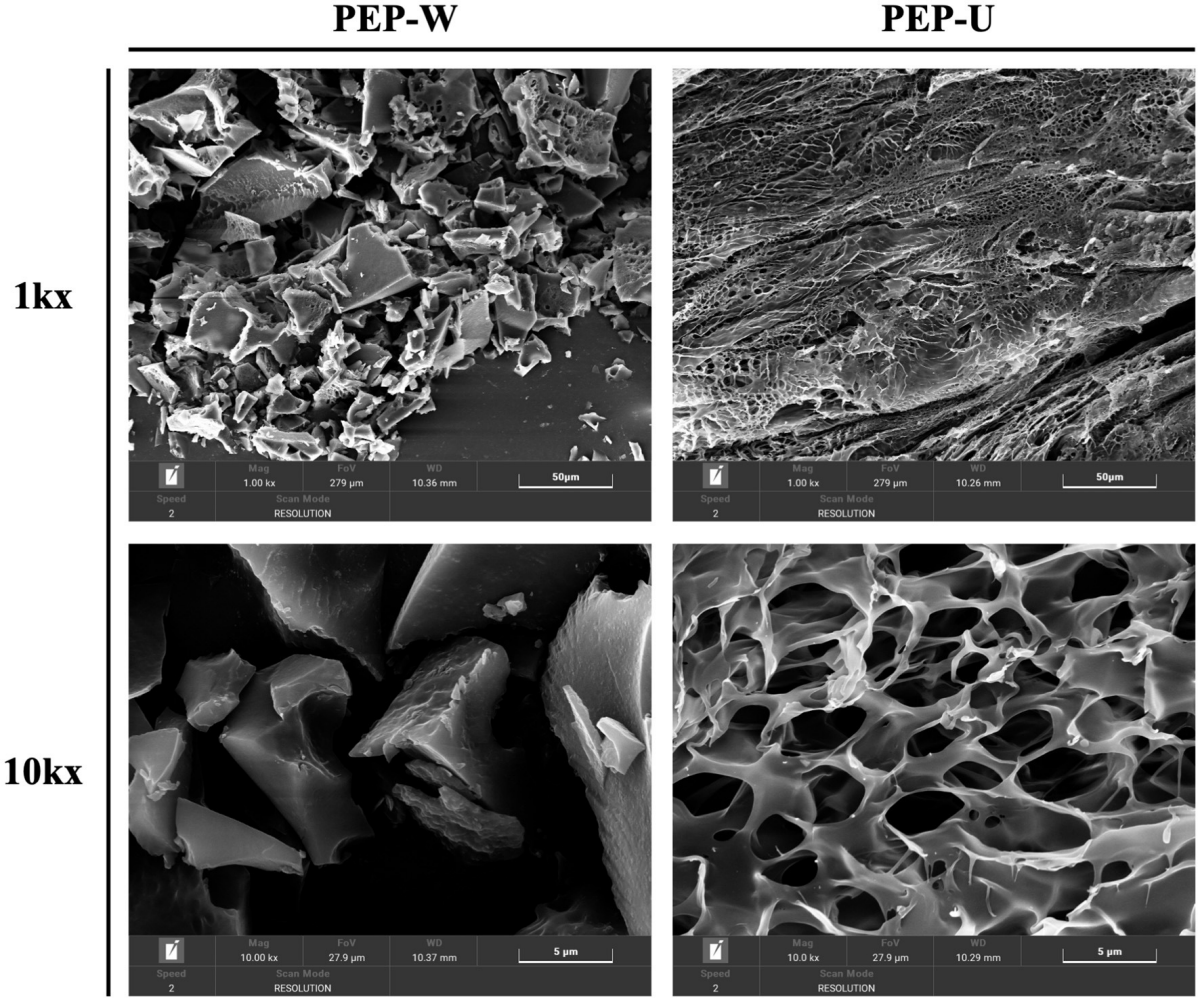
SEM images of PEP-W and PEP-U (1,000×, 10,000×).

Figure 4 presents the scanning electron microscopy (SEM) images of PEP-W and PEP-U, revealing distinct morphological characteristics. The surface of PEP-W is characterized by a relatively smooth appearance with aggregated structures and larger particle sizes, accompanied by minimal porosity or surface cracks. This phenotype is largely due to the absence of external physical forces during the hot water extraction process, which typically results in tighter intermolecular aggregation among polysaccharides, leading to larger particles and a reduced presence of surface defects such as cracks or pores. Conversely, PEP-U, which was extracted using a combination of ultrasonic and microwave treatments, exhibits a more pronounced disruption of the cell wall. This enhanced extraction method leads to a polysaccharide structure with a higher degree of fragmentation and porosity. Therefore, it would be clear that there will be strong intermolecular forces between the glycans under the hot water extraction method, which may inhibit the formation of pores and cell wall disruption. PEP-U, on the other hand, showed a more pronounced cell wall disruption and porosified structure closely related to the mechanical and thermal effects of the combined ultrasound and microwave treatment.

### 3.7 Analysis of antioxidant activity results

#### 3.7.1 Analysis of DPPH radical scavenging activity

**Figure 5:**
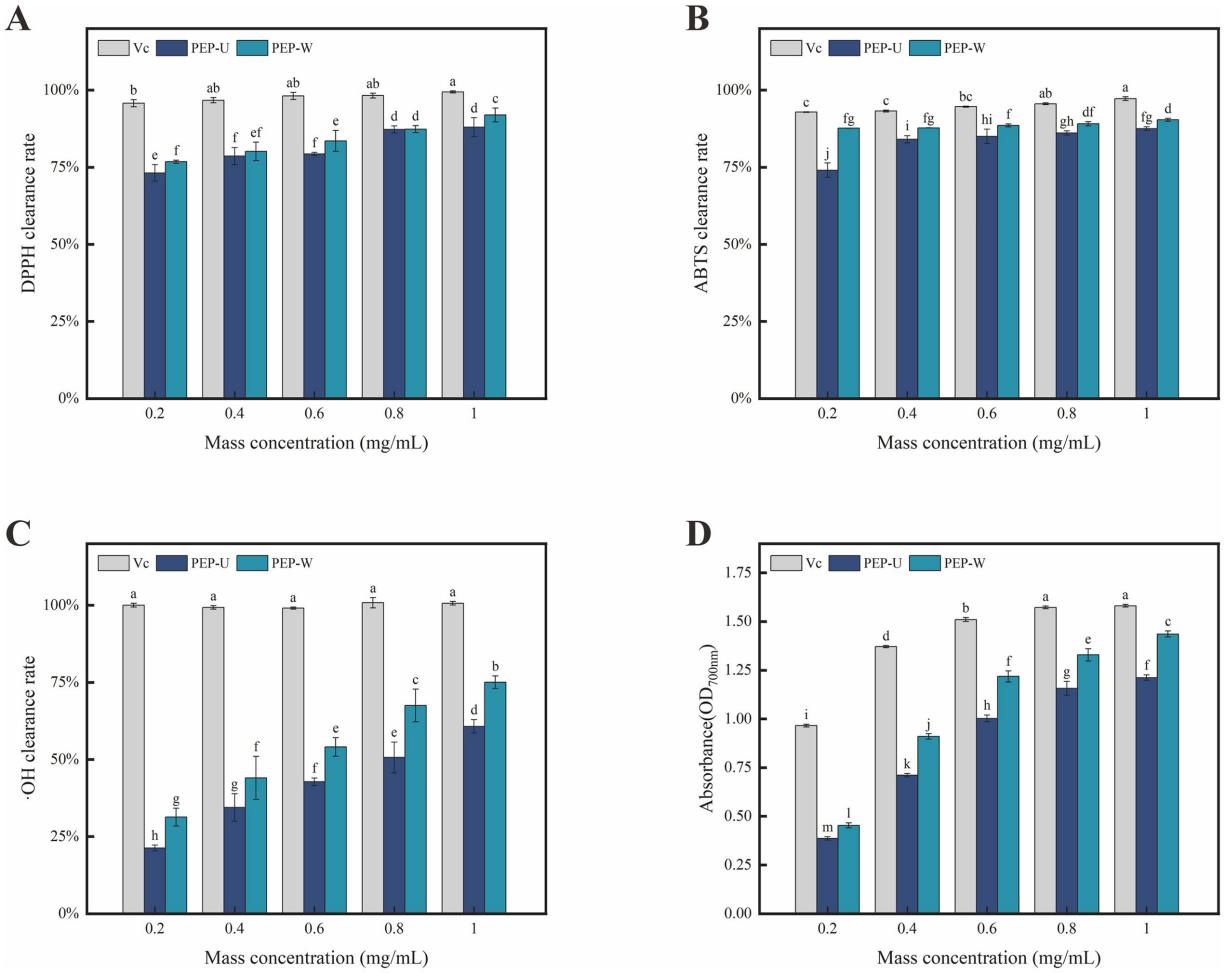
Results of antioxidant activity measurements of Vc, PEP-U and PEP-W. **(A)** DPPH radical scavenging capacity. **(B)** ABTS radical scavenging capacity. **(C)** Hydroxyl radical scavenging capacity. **(D)** Total reducing capacity. Different letters indicate statistically significant differences (*p* < 0.05) among different groups.

The DPPH radical, a stable free radical with a characteristic absorption band at 517 nm in visible spectroscopy, serves as a benchmark for assessing the antioxidant potential of compounds (65). This absorption is markedly reduced upon interaction with a radical scavenger, which neutralizes the DPPH's free radical nature by donating either an electron or a hydrogen atom. The DPPH radical scavenging activity is widely used to assess the antioxidant capacity of polysaccharides. As depicted in Figure 5A, the DPPH radical scavenging efficacy of PEP-U and PEP-W was pronounced within the concentration range of 0.2–1.0 mg/mL, with an incremental increased in activity corresponding to higher concentration. At a concentration of 1.0 mg/mL, PEP-U demonstrated a scavenging rate reached 88.06%, while PEP-W achieved a clearance rate of 91.95%. The superior clearance ability of PEP-W is hypothesized to stem from the mild conditions typically associated with water extraction, which are conducive to preserving the polysaccharides' native molecular weight and structure, thereby maintaining their high scavenging capacity (66). There are also a number of plant polysaccharides such as guara fruits (67) and ginseng (68) that have the ability to scavenge DPPH free radicals, but the scavenging abilities of PEP-U and PEP-W are much higher.

#### 3.7.2 Analysis of ABTS radical scavenging activity

The ABTS radical scavenging assay is a highly sensitive method for detecting antioxidant compounds in samples, as shown in Figure 5B. The scavenging ability of PEP-U and PEP-W against ABTS radicals exhibited a positive correlation with increasing concentration within the range of 0.2–1.0 mg/mL, a trend that closely mirrored that of the positive control, Vitamin C. Conversely, the application of UMSE can substantially disrupt the chemical bonds within polysaccharide molecules, potentially altering their weight and structure. Such alterations may adversely affect the antioxidant capacity of the polysaccharides. This suggests that the extraction method can significantly influence the preservation of antioxidant properties in polysaccharide extracts (69). These findings are consistent with the results of other studies on polysaccharides (70), who found that ultrasonic-assisted extraction improved the antioxidant activity of polysaccharides from *Lycium barbarum* by enhancing the release of active moieties.

#### 3.7.3 Analysis of hydroxyl radical scavenging activity

Hydroxyl radicals are known as a very important reactive oxygen species (ROS) (71), and increasing body of experimental evidence has established their correlation with the process of human aging (72). Consequently, the development of antioxidant substances capable of scavenging hydroxyl radicals and maintaining their levels within a physiologically appropriate range has become a focal point of research (73). Polysaccharides, particularly those derived from medicinal herbs, have garnered considerable attention due to their demonstrated antioxidant capabilities (74), such as Ganoderma lucidum polysaccharides (75). As shown in Figure 5C, both PEP-U and PEP-W exhibit scavenging activity against hydroxyl radicals, albeit with efficacy that is somewhat lower than that of the positive control. At a concentration of 1.0 mg/mL, PEP-U achieved a scavenging rate of 60.79%, while PEP-W reached a clearance rate of 75.07%. The upward trend in their scavenging activity suggests that both polysaccharides may continue to show significant increases in efficacy beyond the maximum tested concentration of 1.0 mg/mL.

#### 3.7.4 Reducing power assay

The total reducing power is a pivotal parameter for assessing the overall antioxidant capacity of a sample (76). The mechanism of the reducing agent is to prevent the propagation of chain reaction by scavenging free radicals, and its principle is mainly to reduce potassium ferricyanide to potassium ferrocyanide through the reducing ability of the sample (77), which can be quantitatively evaluated by measuring the changes of absorbance (78). As shown in Figure 5D, the absorbance of the solution for both PEP-U and PEP-W escalated progressively within the concentration range of 0.2–1.0 mg/mL. This increment in absorbance corresponds to an escalating reducing ability as the concentration of the polysaccharides increases. These findings suggest that both PEP-U and PEP-W possess potential as natural antioxidant sources, with their reducing power enhancing in tandem with concentration.

### 3.8 Simulated *in vitro* digestion of PEP

#### 3.8.1 Effect of artificial saliva on polysaccharide hydrolysis

**Figure 6:**
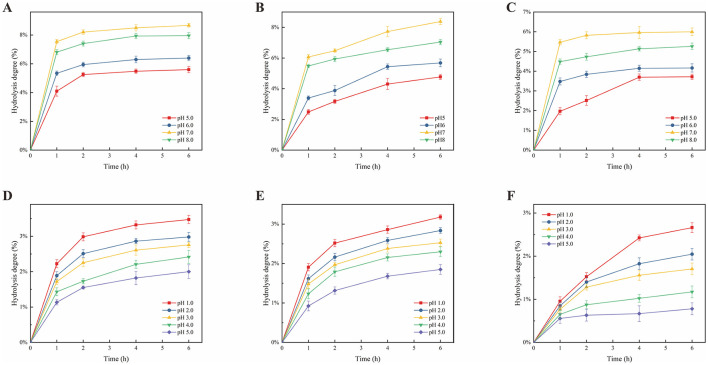
Effect of artificial saliva and artificial gastric fluid on polysaccharide hydrolysis. **(A)** Effect of artificial saliva on FOS hydrolysis. **(B)** Effect of artificial saliva on PEP-U hydrolysis. **(C)** Effect of artificial saliva on PEP-W hydrolysis. **(D)** Effect of artificial gastric fluid on FOS hydrolysis. **(E)** Effect of artificial gastric fluid on PEP-U hydrolysis. **(F)** Effect of artificial gastric fluid on PEP-W hydrolysis.

As prebiotics intended for human consumption (79), polysaccharides should resist hydrolysis by saliva, traverse the gastrointestinal tract without degradation, and modulate the probiotic population in the gastrointestinal tract. The hydrolysis profiles of PEP-U and PEP-W over time are presented in Figures 6A–C. Both PEP-U and PEP-W exhibited greater resistance to hydrolysis than the positive control FOS. After a 6-h of incubation at pH 5, 6, 7, and 8, the hydrolysis degrees for PEP-W were 3.72%, 4.16%, 5.99%, and 5.26%, respectively, while those for PEP-U were 4.77%, 5.68%, 8.38%, and 7.04%. The hydrolysis degree of polysaccharides was significantly affected by pH, with the impact decreasing in the order of pH 7 > 8 > 6 > 5. This susceptibility to pH may be attributed to the presence of amylase in saliva, which plays a crucial role in polysaccharides hydrolysis. The enzymatic activity of amylase is pH-dependent (80), with an optimal pH range of 6.7–7.0 (81), where it is most stable and active. At pH 7, the enzyme's activity is at its peak, resulting in the highest hydrolysis degree. Conversely, at pH 5, which is considerably lower than the optimal pH, the enzyme's structure and function are more compromised, leading to a significantly decrease in activity and the lowest hydrolysis degree (82). Furthermore, the hydrolysis of polysaccharides is intricately linked to their sugar structure (83), composition, and configuration, including ring shape and chemical bonding (84). PEP-U, extracted using microwave and ultrasonic methods, likely has a more complex polysaccharide structure with more intense chemical bond breakage compared to the water extraction method used for PEP-W (85). This complexity may contribute to a higher degree of hydrolysis for PEP-U.

#### 3.8.2 Effect of artificial gastric juice on polysaccharide hydrolysis

In general, food usually reaches the stomach within a 4 h timeframe. The gastric fluid was used to assess the hydrolytic resistance of PEP-U and PEP-W. The hydrolysis of PEP-U and PEP-W by gastric juice is shown in Figures 6D–F. Following a 6 h incubation at pH 1, 2, 3, 4, and 5, the hydrolysis percentages for PEP-W were 2.66%, 2.05%, 1.70%, 1.17%, and 0.78%, respectively, while those for PEP-U were 3.18%, 2.84%, 2.53%, 2.30%, and 1.85%. The results showed that PEP-U and PEP-W exhibit over 95% resistance to gastric juice, with maximum hydrolysis degrees of 3.18%, 2.66%, and 3.47% for PEP-U, PEP-W and FOS, respectively. This suggests that both polysaccharides maintain good stability under acidic conditions and low temperatures, with minimal variation in their resistance to human gastric juice digestion, outperforming FOS. The influence of pH on hydrolysis degree was observed in descending order as 1 > 2 > 3 > 4 > 5. Hydrochloric acid (86), the predominant component in gastric juice, is capable of hydrolyzing the glycosidic bonds within polysaccharide molecules, particularly α-1,4 and α-1,6 glycosidic bonds (87). The hydrolytic capacity is enhanced at lower pH value, with efficiency diminishing as pH increase, resulting in a corresponding decrease in hydrolysis degree. Furthermore, the duration of incubation impacts the hydrolysis of polysaccharides, as extended incubation periods can leads to the degradation of polysaccharides into monosaccharides and disaccharides (88). Consequently, it can be inferred that the majority of PEP-U and PEP-W can traverse the stomach and reach the intestine, where they can be utilized by the probiotic populations residing therein.

### 3.9 Effect of polysaccharides on the growth of probiotics

#### 3.9.1 Analysis of the effect of polysaccharides on the growth curves of probiotics at optimal addition levels

**Figure 7:**
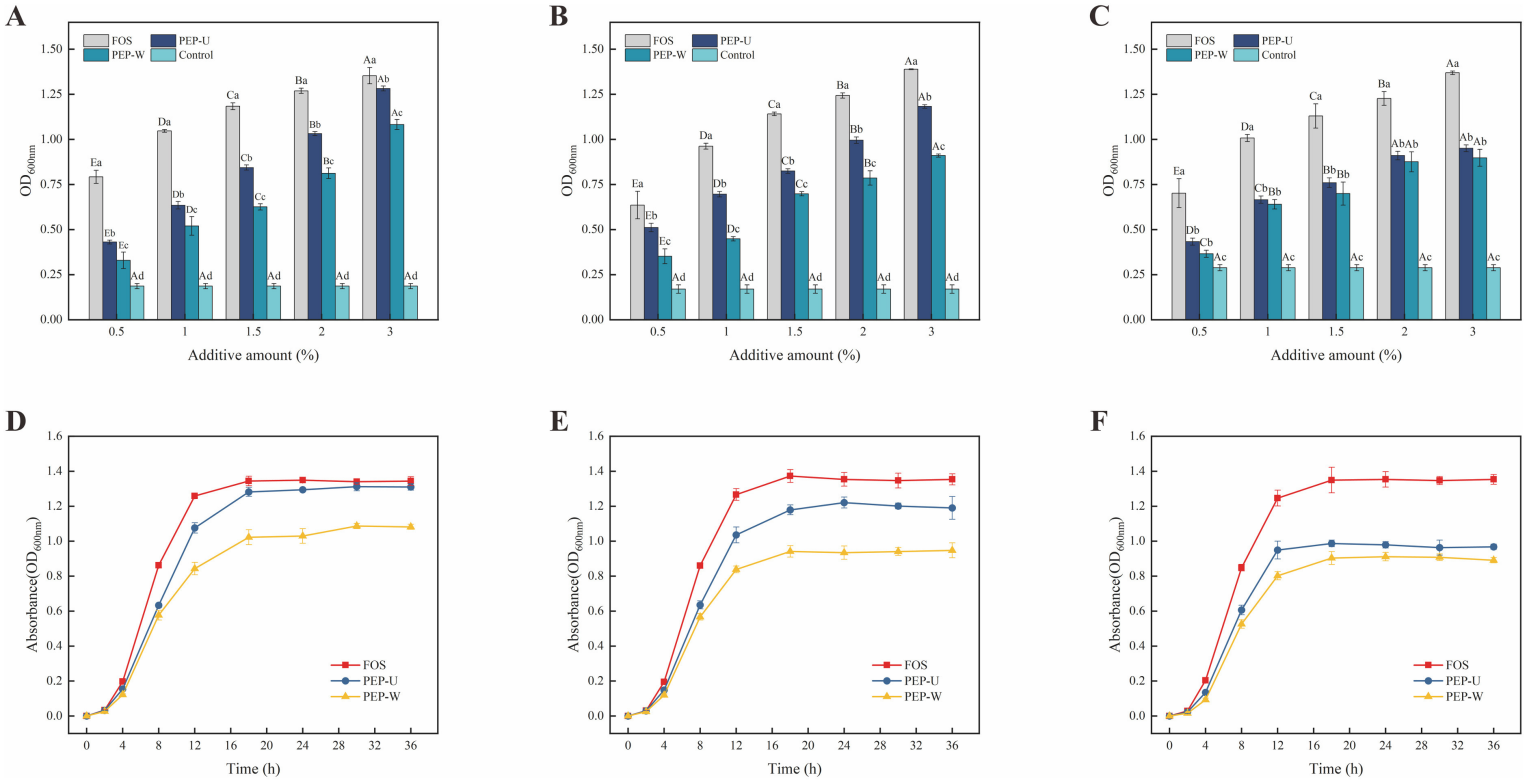
Optimal addition of polysaccharides explored with probiotic growth curves at optimal addition. **(A)** Optimal addition of polysaccharides for *Lactobacillus casei*. **(B)** Optimal addition of polysaccharides for *Lactobacillus acidophilus*. **(C)** Optimal addition of polysaccharides for *Lactobacillus plantarum*. **(D)** Growth curve of *Lactobacillus casei*. **(E)** Growth curve of *Lactobacillus acidophilus*. **(F)** Growth curve of *Lactobacillus plantarum*.

The optical density at 600 nm (OD_600nm_) was employed as an indicator of probiotic growth, with higher OD_600nm_ values correlating to better growth conditions at a given polysaccharide concentration. The prebiotic effects of varying concentrations of polysaccharides on three probiotic species are illustrated in Figures 7A–C. For *Lactobacillus casei* and *Lactobacillus acidophilus*, the optimal polysaccharide concentration was determined to be 3%, at which the OD_600nm_ value of PEP-U was approximated that of the positive control FOS, following a 36 h incubation period. In the case of *Lactobacillus plantarum*, no significant difference in OD_600nm_ values was observed between 2% and 3% polysaccharide supplementation. Taking into account economic costs and other relevant factors, the optimal concentration for *Lactobacillus plantarum* was selected to be 2%.

The growth curves of three probiotic strains were constructed using PEP-U, PEP-W, and FOS at the respective optimal concentrations to serve as carbon sources probiotic proliferation, as depicted in Figures 7D–F. The analysis reveals that both PEP-U and PEP-W can promote the proliferation of the three probiotics, with a more pronounced effect on *Lactobacillus casei* and a comparatively weaker effect on *Lactobacillus plantarum*. The order of proliferative effects of PEP-U and PEP-W on the probiotics from strongest to weakest is *Lactobacillus casei* > *Lactobacillus acidophilus* > *Lactobacillus plantarum*. The stimulatory effect of PEP-U on *Lactobacillus casei* was comparable to that of the positive control, FOS, indicating that PEP-U was more readily assimilated and utilized by *Lactobacillus casei*, thereby exhibiting a favorable prebiotic effect. The growth curves indicate distinct phases: an initial lag phase from 0 to 2 h, during which the probiotics adapt to the environment; a logarithmic growth phase from 2 to 18 h, as the probiotics absorb nutrients and proliferate; and a stationary phase from 18 to 36 h, characterized by the depletion of nutrients and a consequent decline in growth rate. Notably, PEP-U showed a more significant proliferative effect than PEP-W, despite the general trend that indigestible carbohydrates with lower molecular weights tend to have higher proliferative effects. The lower proliferative effects of PEP-U and PEP-W compared to FOS are likely due to FOS's smaller molecular weight, aligning with Li's findings that bifidobacteria preferentially utilize short-chained FOS and inulin (89). Furthermore, polysaccharides with more branched chains had a stronger prebiotic effect (90), and PEP-U had a stronger proliferative effect, possibly due to the increased complexity and branching.

The strong prebiotic activity exhibited by PEP-U significantly promoted the growth of the three Lactic Acid Bacteria. The molecular mechanism behind this may be closely related to the polysaccharide composition of PEP-U. Studies have shown that the polysaccharides can promote intracellular nutrient uptake by enhancing the permeability of the cell membrane of Lactic Acid Bacteria (91). Polysaccharides also promote the growth of Lactic Acid Bacteria by improving their antioxidant capacity and enhancing their tolerance to oxidative stress (92). Further studies suggest that specific sugar units such as galacturonic acid in PEP-U may promote the utilization of carbon sources and energy production by modulating the metabolic pathways of Lactic Acid Bacteria, which may also be one of the promotional effects of PEP-U on the growth of Lactic Acid Bacteria (93). Overall, PEP-U might significantly enhance the growth performance of Lactic Acid Bacteria through a multi-pathway molecular mechanism, which proves its application as a potential prebiotic.

#### 3.9.2 Changes in lactate content and pH during proliferation

**Figure 8:**
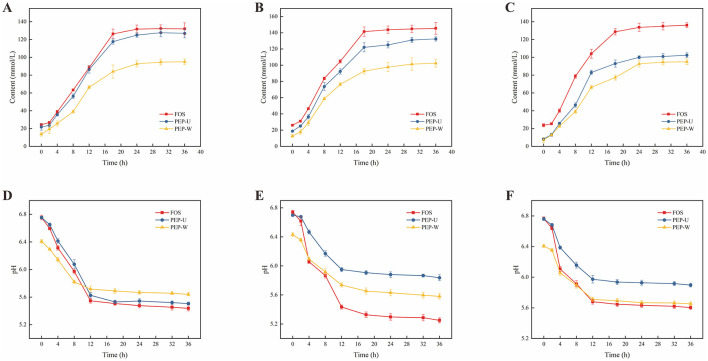
Changes in lactic acid content and dynamics of pH during the culture of probiotics. **(A)** Changes in the content of lactic acid during the culture of *Lactobacillus casei*. **(B)** Change in the content of lactic acid during the culture of *Lactobacillus acidophilus*. **(C)** Changes in lactic acid content during culture of *Lactobacillus plantarum*. **(D)** Changes in pH during culture of *Lactobacillus casei*. **(E)** Change in pH during cultivation of *Lactobacillus acidophilus*. **(F)** Change in pH during cultivation of *Lactobacillus plantarum*.

The dynamics of lactic acid production by three probiotic strains during incubation are delineated in Figures 8A–C. Over the initial 0–36 h, lactic acid levels in all three cultures escalated progressively, likely attributable to the ongoing consumption of polysaccharides and concomitant lactic acid production by lactobacilli as they grew (94). After 18 h, the rate of change in lactic acid content was diminished, potentially due to the probiotic bacteria transitioning into a stationary phase characterized by nutrient depletion and a consequent deceleration in bacterial growth, which in turn slowed lactic acid accumulation.

Polysaccharides, serving as a carbon source, are metabolized by probiotics, yielding short-chain fatty acids and causing a concomitant decrease in culture pH (95). Monitoring these pH fluctuations provides an index of probiotic activity (96). The pH changes are shown in Figures 8D–F. During the first 18 h, a precipitous drop in pH was observed, a result of the probiotics' polysaccharide metabolism and lactic acid production. Beyond 18 h, as the carbon source in the culture was depleted and probiotic growth slowed, the production of short-chain fatty acids also subsided, leading to a stabilization of pH levels.

## 4 Discussion

Polysaccharides were successfully extracted from *Phyllanthus emblica* L. using ultrasonic microwave synergistic extraction (UMSE), and the process was optimized through single-factor experiments and response surface methodology (RSM). The optimized conditions significantly improved extraction efficiency and purity compared with traditional hot water extraction, resulting in a yield of 8.09%, consistent with predicted values. Both UMSE-extracted (PEP-U) and water-extracted (PEP-W) polysaccharides exhibited strong antioxidant activities and high resistance to hydrolysis in simulated digestion. Additionally, both polysaccharides promoted the growth of *Lactobacillus* species, with PEP-U showing a more pronounced effect, likely due to structural differences.

These results indicate that *Phyllanthus emblica* L. polysaccharides, particularly those obtained via UMSE, possess promising antioxidant and prebiotic potential, supporting their application in functional foods and nutraceuticals. However, some limitations should be considered when interpreting the findings. First, the extraction efficiency and yield may vary depending on the raw material's source and quality, which could influence the reproducibility of the results across different batches. Additionally, while the study demonstrated the prebiotic effects of the polysaccharides, the mechanisms underlying their interactions with specific gut microbiota are not yet fully understood and warrant further investigation.

Looking ahead, there is significant potential for further exploration. Future studies could focus on characterizing the polysaccharides' structural features, such as molecular weight distribution and glycosidic linkage patterns, to better understand their functional properties. Moreover, *in vivo* models could provide valuable insights into the bioavailability and systemic effects of these polysaccharides, further solidifying their potential for human health. Exploring the polysaccharides' interaction with the gut microbiome and their broader impact on gut health would also be an exciting avenue for future research.

## 5 Conclusion

In this study, polysaccharides were successfully extracted from *Phyllanthus emblica* L. using an ultrasonic microwave synergistic extraction (UMSE) method, and the extraction conditions were optimized through single-factor experiments combined with response surface methodology (RSM). The optimized parameters—microwave power of 370 W, ultrasound power of 340 W, extraction time of 25 min, and a material-to-liquid ratio of 1:6.5 g/mL—resulted in a polysaccharide yield of 8.09%, closely matching the predicted value. Compared with traditional hot water extraction, UMSE significantly enhanced both extraction efficiency and polysaccharide purity.

Antioxidant assays, including DPPH, ABTS, hydroxyl radical scavenging, and total reducing power, demonstrated that both UMSE-extracted (PEP-U) and water-extracted (PEP-W) polysaccharides possess potent antioxidant activities. Simulated digestion tests showed that both polysaccharides exhibited strong resistance to hydrolysis in artificial saliva and gastric juice, with retention rates exceeding 95%, suggesting their stability through the upper digestive tract. Prebiotic potential was confirmed by the enhanced growth of probiotic strains, with PEP-U showing superior stimulatory effects—likely due to its more complex structural features.

Overall, these findings indicate that *Phyllanthus emblica* L. polysaccharides, particularly those obtained via UMSE, hold strong potential for application as natural antioxidants and novel prebiotic ingredients in the development of functional foods, gut health supplements, and nutraceutical formulations. Future studies should focus on *in vivo* validation of prebiotic efficacy, structure–activity relationship analysis, and formulation development to facilitate their commercial use in the food and health industries.

## Data Availability

The raw data supporting the conclusions of this article will be made available by the authors, without undue reservation.
